# Genetic Association Between *PER3* Genetic Polymorphisms and Cancer Susceptibility: A Meta-Analysis

**DOI:** 10.1097/MD.0000000000000568

**Published:** 2015-04-03

**Authors:** Peiliang Geng, Juanjuan Ou, Jianjun Li, Ning Wang, Ganfeng Xie, Rina Sa, Chen Liu, Lisha Xiang, Houjie Liang

**Affiliations:** From the Department of Oncology and Southwest Cancer Center, Southwest Hospital, Third Military Medical University, Chongqing, China.

## Abstract

The genes along the circadian pathways control and modulate circadian rhythms essential for the maintenance of physiological homeostasis through self-sustained transcription-translation feedback loops. *PER3* (period 3) is a circadian pathway gene and its variants (rs1012477, 4/5-repeat) have frequently been associated with human cancer. The mixed findings, however, make the role of the 2 variants in cancer susceptibility elusive. We aimed in this article to clarify the association of *PER3* variants with cancer.

We collected genetic data from 8 studies, providing 6149 individuals for rs1012477 and 5241 individuals for 4/5-repeat. Based on the genotype and allele frequency, we chose the fixed-effects model to estimate risk of cancer.

Overall analysis did not suggest a global role of rs1012477 in cancer susceptibility. For *PER3* 4/5-repeat variant, we found a moderate increase in risk of cancer among individuals with the 5-allele compared to individuals with the 4-allele, although this association was not statistically significant (homozygous model: odds ratio [OR] 1.17, 95% confidence interval [CI] 0.81–1.67; recessive model: OR 1.17, 95% CI 0.82–1.67). No substantial heterogeneity was revealed in this analysis.

Our meta-analysis provides no evidence supporting a global association of *PER3* genetic variants with the incidence of cancer.

## INTRODUCTION

Circadian clock represents a fundamental biological system responsible for the mediation of periodic physiological and behavioral change. A handful of work in the past decade has well defined the molecular mechanisms underlying the complex multioscillatory temporal network that functions efficiently in several biochemical processes including cell cycle and carcinogen metabolism.^1–3^ Circadian pathway genes involved in regulating multiple cancer-related pathways, like DNA damage and repair, cell growth, and cell death, are usually biologically determined by light-night alternations.^4^ Detrimental mutations in the genes along the circadian pathways, together with exposure to carcinogenic substances possibly cause dysfunction of initially intact cells, and a subsequent increase in the risk of cancer.^5^ Investigating the molecular function of circadian genes in human carcinogenesis therefore seems to be helpful in identifying individuals at higher risk of cancer.

*PER3* (period 3) is one of many core circadian genes that maintain circadian rhythms in a normal condition through transcriptional-translational feedback loops where both positive and negative activators, such as *PER3*, are involved.^6^ LAN (light at night)-related disruption of the circadian rhythm has previously been focused and been presumed to facilitate tumorigenesis via suppressing antiproliferative activity of nocturnal melatonin signal.^7–9^ Increased incidence of prostate, breast, endometrial, and colorectal cancer has been reported in several groups of patients who have shift work.^10–13^ Another 2 periods, *PER1* and *PER2*, are described in expression-based studies to be lowly expressed in glioma cell lines relative to the adjacent benign cell lines.^14^ Given the potential effects of *PER1* and *PER2* on carcinogenesis, it has been hypothesized that *PER3*, its genetic variants in particular, may modulate inherited susceptibility to human cancer.

*PER3* single-nucleotide polymorphism (SNP) rs1012477 was first validated as a cancer susceptibility locus in a population-based case-control study of white men,^15^ with most of the replications reporting substantially different findings.^16,17^ As for 4/5-repeat, a majority of the individually published studies did not suggest a causative association with cancer.^18–20^ To assess the true association between the 2 *PER3* SNPs and cancer susceptibility, we for the first time combined all different single studies and performed a comprehensive meta-analysis.

## METHODS

### Literature Search

A thorough literature search was undertaken using the Embase (http://www.embase.com), PubMed (http://www.ncbi.nlm.nih.gov/pubmed), and Science Direct (http://www.sciencedirect.com) databases, with the last search conducted on December 1, 2013. We used search terminology (polymorphism or polymorphisms) AND (period 3) AND (cancer) and their synonyms (variants, genotypes, *PER3*) to identify all possibly relevant studies. In addition, to obtain novel data, 2 authors hand searched references of the articles considered eligible in this meta-analysis. The study was approved by the ethics committee of southwest hospital of third military medical university.

### Inclusion Criteria and Data Abstraction

All association studies eligible for this meta-analysis were required to meet all of the following inclusion criteria:

1. A human study with a case-control or cohort design;
2. Examining the correlation between at least one of the *PER3* genetic polymorphisms of interest and cancer incidence;
3. Providing detailed genotype counts essential for the calculation of odds ratios (OR) and 95% confidence intervals (CI).

To maximize the accuracy of extracted data, the authors hand searching the references of all eligible studies separately collected authors’ names, date when the paper was published, patients and controls included in each analysis, ethnicity (white or Asian descent), *P* value for Hardy–Weinberg equilibrium (HWE) if available, type of cancer, study design (retrospective or prospective), source of controls (hospital or population based), genetic data, and polymorphism studied. If more than 1 paper pertaining to the same topic included duplicate samples, we considered the largest study with the most informative and complete data.

### Statistics

Using the genotype and allele frequencies in cases and controls, we estimated risk of cancer (OR and 95% CI) associated with *PER3* polymorphisms. ORs were summarized under homozygous, heterogeneous, dominant, recessive, and allele models for both variants. The Z test was used to test the significance of all pooled ORs and a *P* value smaller than 5% was considered significant.

The calculation of summary ORs was undertaken using a fixed-effects (Mantel–Haenszel method) when the studies included were homogeneous (*P* > 0.05) and a random-effects (DerSimonian–Laird) when apparent heterogeneity was indicated (*P* ≤ 0.05). Between-study heterogeneity was detected using Cochran Q statistic. Visual inspection of the funnel plots and Egger test were utilized to determine potential publication bias in the literature.^21^ HWE was verified in controls using the goodness-of-fit χ^2^ test. Sensitivity analysis was used to test the validity of combined results. Meta-analysis was conducted with stata 12.0 (Stata, College Station, TX). The 2-tailed *P* values were determined to be significant at 0.05.

## RESULTS

### Selection Process and Study Characteristics

**FIGURE 1:**
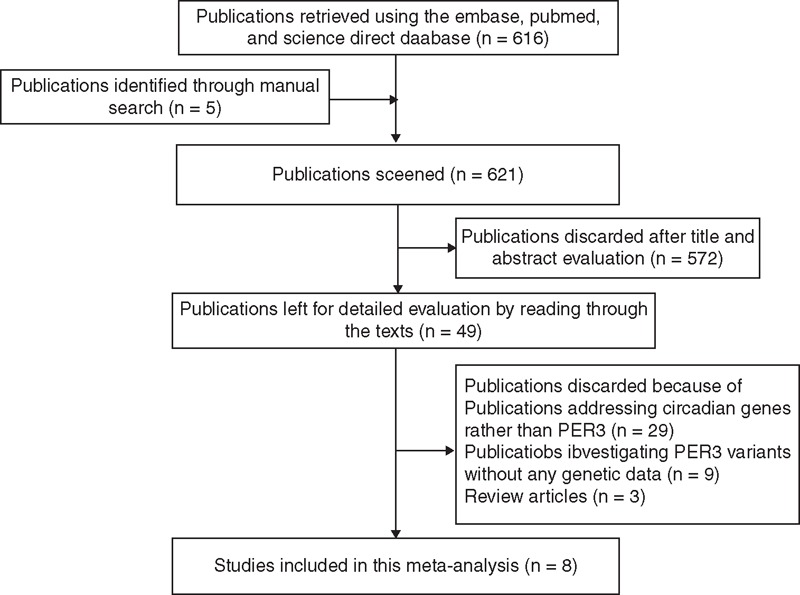
A Flow chart summarizing the study selection process. *PER3* =  period 3.

Figure 1 summarizes the reasons of excluding/including studies. We first evaluated the title and abstract of all 621 retrieved records, and discarded 572 expression- or survival-based and nonhuman studies. We were left with 49 records and then read through the texts. A total of 41 papers were further excluded, because they addressed circadian genes rather than *PER3*, investigated *PER3* variants without any genetic data, or published as review articles. After excluding all ineligible records, we were finally left with 8 retrospective studies.^15–20,22,23^

As described in Table 1, 4 studies had investigated *PER3* rs1012477 and all samples were whites. Three types of malignant diseases, such as prostate cancer, colorectal cancer, and glioma were combined. Of the *PER3* 4/5-repeat studies, both Asian and white samples were employed. Neither of the white studies was in accord with HWE. Cancer of prostate, breast, and colorectal was studied in the publications of *PER3* 4/5-repeat.

**TABLE 1:**
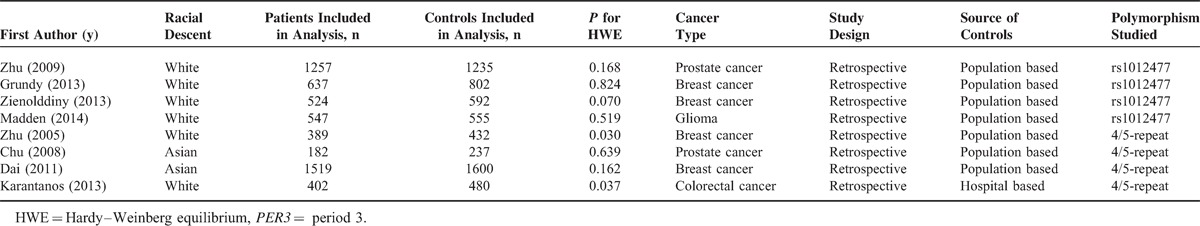
Characteristics of Studies on *PER3* Genetic Variants and Cancer Patients

### Quantitative Analysis

**FIGURE 2:**
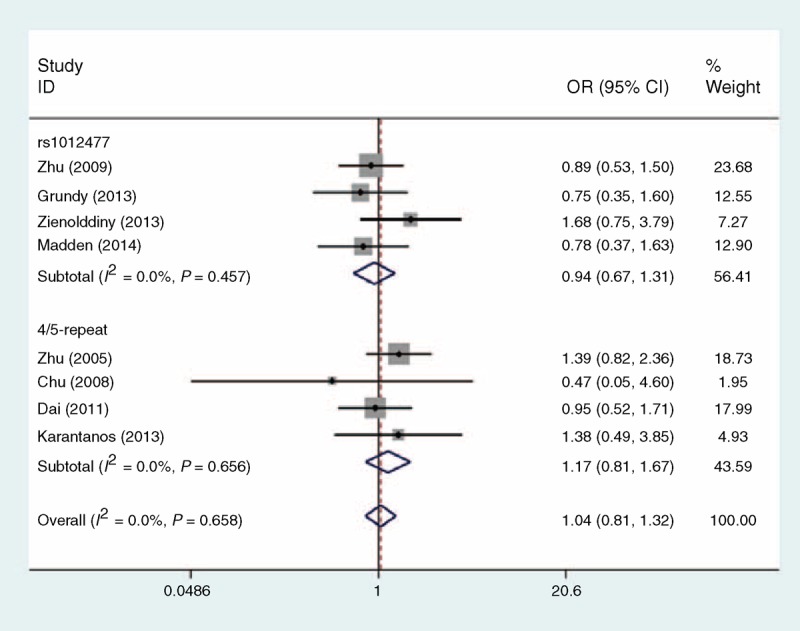
Meta-analysis for *PER3* genetic variants and cancer susceptibility under the homozygous model. Each study was shown by a point estimate of the effect size (OR) (size inversely proportional to its variance) and its 95% confidence interval (95% CI) (horizontal lines). The diamonds represent the pooled ORs. CI = confidence interval, OR = odds ratio, *PER3* = period 3.

**FIGURE 3:**
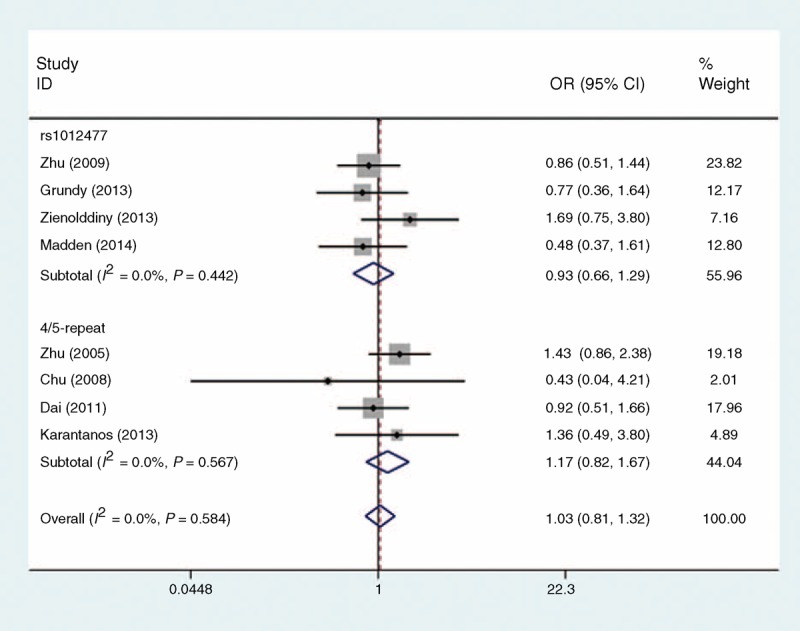
Meta-analysis for *PER3* genetic variants and cancer susceptibility under the recessive model. Each study was shown by a point estimate of the effect size (OR) (size inversely proportional to its variance) and its 95% confidence interval (95% CI) (horizontal lines). The diamonds represent the pooled ORs. CI = confidence interval, OR = odds ratio, *PER3* = period 3.

Table 2 shows the ORs and 95% CIs for all models tested in this analysis. Based on 2965 cases and 3184 controls for rs1012477, the overall analysis did not show statistical evidence of a significant association between rs1012477 and cancer risk in any of the genetic models tested: homozygous model (GG vs CC: OR 0.94, 95% CI 0.67–1.31, Figure 2), heterogeneous model (CG vs CC: OR 1.02, 95% CI 0.92–1.14), dominant model (GG + CG vs CC: OR 1.02, 95% CI 0.91–1.13), recessive model (GG vs CG + CC: OR 0.93, 95% CI 0.66–1.29, Figure 3), allele model (G vs C: OR 1.01, 95% CI 0.92–1.11).

**TABLE 2:**
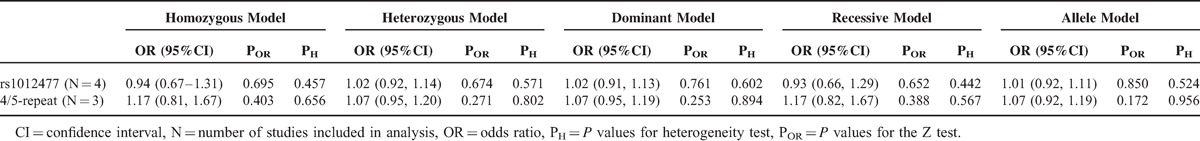
Meta-Analysis of All Studies and Subgroups

Three eligible studies of *PER3* 4/5-repeat yielded a total of 2492 cancer patients and 2749 noncancerous controls. We performed an analysis in total samples and found individuals with the 5-allele had 17% increased risk of cancer compared to individuals with the 4-allele, although this association was not statistically significant (homozygous model: OR 1.17, 95% CI 0.81–1.67, Figure 2; recessive model: OR 1.17, 95% CI 0.82–1.67, Figure 3).

Removing the single studies one by one did not show any obvious change in the combined effect size estimates, suggesting our results were statistically robust and reliable. According to the funnel plots (available upon request) and Egger test, there was no significant publication bias in this meta-analysis (allele model: *P* = 0.196 for *PER3* rs1012477, *P* = 0.256 for *PER3* 4/5-repeat).

## DISCUSSION

*PER3* plays a major role in tumor suppression and alternations in gene expression levels have been associated with incidence of cancer.^24,25^ The first molecular epidemiological analysis of rs1012477 and prostate cancer provided statistical evidence that men with the G allele, compared to men with the C allele, had around 25% higher risk of prostate cancer.^15^ Grundy afterward associated this variant with breast cancer and found no connection in a population of white origin,^16^ a finding in agreement with a recent replication.^22^ Interestingly, in a group of white women, authors observed that the variant CG genotype compared with the CC genotype was linked with an almost 58% lower risk of breast cancer for women with 3 consecutive night shifts, while no association was implicated in those with less than 2 or at least 4 consecutive nights.^17^ The wide divergence in these observations can be explained by the differences in ethnic origin, clinical characteristics (eg, duration of shift work), adjusted factors, and samples in each of the published studies.

The 4/5-repeat variant has also received close attention in recent years. Several lines of evidence have shown this functional variant does not appear to play a significant role in the development of various cancers including aggressive prostate, breast, and colorectal cancer.^18–20^ It is interesting that the first study in an American population has reported a significantly increased risk of breast cancer among premenopausal women.^23^ The significance, however, was lost in a later larger replication in Chinese samples.^19^ It is possible that Zhu et al have demonstrated a false-positive finding most likely due to the relatively small number of individuals and reached a biased conclusion as a consequence.

As the single studies may have inadequate statistical power to precisely assess the effects of *PER3* genetic variants on cancer susceptibility, we carried out a meta-analysis known as a quantitative approach to maximize detection power overall with an aim to provide compelling evidence for the association between SNPs and malignant diseases. We included 6149 individuals for rs1012477 and 5241 individuals for 4/5-repeat in this article. Meta-analysis of rs1012477 suggested no associations with cancer susceptibility. A similar pattern was found for 4/5-repeat, which is consistent with most of the published studies on the association between 4/5-repeat and cancer.^18–20^ Since significant impact of rs1012477 and 4/5-repeat on the progression of cancer has been reported in several studies,^15,17,23^ we cannot exclude the etiologic significance of these molecular variants in cancer susceptibility and it seems that only a substantially large study can detect the minor effects of the low-penetrance SNPs.

Cellular circadian system is quite a complex biological network. The system is further complicated by Casein kinase 1 ε that serves as a mediator of PER at a posttranslational level and makes the proteins aberrantly expressed via phosphorylation; significantly lower expression of the *PER3* gene has been detected in cancer tissue relative to the adjacent normal mucosa.^26^ There is abundant evidence supporting a causative connection between degradation of *PER3* and human carcinogenesis. Climent et al demonstrated recently that *PER3* is underexpressed in estrogen receptor–positive patients with breast cancer and that loss of *PER3* is closely associated with tumor recurrence, especially in the patients being treated with tamoxifen; moreover, mice with *PER3* deficiency are found to be more susceptible to the invasive cancer.^27^ These data, coupled with the positive associations implicated in epidemiological studies, suggest that genetic variants in the *PER3* gene may be genetic susceptibility biomarkers and play a key role in oncogenesis.

Several studies have identified duration of shift work and menopausal status as covariants in the association of *PER3* variants and incidence of cancer.^17,23^ Therefore, the combination of genetical and environmental factors is worth further exploring in future cancer studies. Our meta-analysis did not reveal a global role of the functional SNPs in cancer susceptibility, but we cannot rule out the possibility that the low-penetrant SNPs modulate risk of some specific type of cancer and only a large-scale analysis can detect the slight effects. The third shortcoming in this article is the potential publication bias. Although we have utilized 2 analytic tools with no evidence of significant bias detected, inclusion of published data only suggests results of the present study have more or less been biased and should be explained with caution.

In summary, our meta-analysis, on the basis of statistical data, suggested no relevance of common variants in *PER3* to cancer susceptibility. Further research, especially prospective studies with a sufficient sample size for each cancer type, is necessary to validate our findings and to clarify whether duration of shift work acts as a cofounding factor for incidence of cancer.
